# Lymphatic outflow of cerebrospinal fluid is reduced in glioma

**DOI:** 10.1038/s41598-019-51373-9

**Published:** 2019-10-15

**Authors:** Qiaoli Ma, Felix Schlegel, Samia B. Bachmann, Hannah Schneider, Yann Decker, Markus Rudin, Michael Weller, Steven T. Proulx, Michael Detmar

**Affiliations:** 1 Institute of Pharmaceutical Sciences, Swiss Federal Institute of Technology, ETH Zurich, Zurich, Switzerland; 2 Institute of Biomedical Engineering, University of Zurich and ETH Zurich, Zurich, Switzerland; 3 Neuroscience Center Zurich, University of Zurich and ETH Zurich, Zurich, Switzerland; 4 Laboratory of Molecular Neuro-Oncology, Department of Neurology, University Hospital and University of Zurich, Zurich, Switzerland; 5 Department of Neurology, University of the Saarland, Homburg, Germany; 6 Institute of Pharmacology and Toxicology, University of Zurich, Zurich, Switzerland; 7 Theodor Kocher Institute, University of Bern, Bern, Switzerland

**Keywords:** Circulation, CNS cancer

## Abstract

Glioblastoma is a malignant brain tumor with mean overall survival of less than 15 months. Blood vessel leakage and peritumoral edema lead to increased intracranial pressure and augment neurological deficits which profoundly decrease the quality of life of glioblastoma patients. It is unknown how the dynamics of cerebrospinal fluid (CSF) turnover are affected during this process. By monitoring the transport of CSF tracers to the systemic blood circulation after infusion into the cisterna magna, we demonstrate that the outflow of CSF is dramatically reduced in glioma-bearing mice. Using a combination of magnetic resonance imaging (MRI) and near-infrared (NIR) imaging, we found that the circulation of CSF tracers was hindered after cisterna magna injection with reduced signals along the exiting cranial nerves and downstream lymph nodes, which represent the major CSF outflow route in mice. Due to blockage of the normal routes of CSF bulk flow within and from the cranial cavity, CSF tracers were redirected into the spinal space. In some mice, impaired CSF clearance from the cranium was compensated by a lymphatic outflow from the sacral spine.

## Introduction

Glioblastoma arises from astrocytes and is classified as World Health Organization Grade 4 infiltrative glioma. The mean overall survival achieved with repeat surgery or irradiation or with salvage chemotherapy remains only 14.6 months^1,2^. Glioblastoma is one of the most vascularized cancers. The newly formed blood vessels are leaky with a disrupted blood-brain-barrier (BBB), which leads to the formation of peritumoral edema and increased intracranial pressure^3^. Neurological symptoms and signs exacerbated by this edema dramatically reduce the life quality of glioblastoma patients^4^.

Cerebrospinal fluid (CSF) is produced mainly by the choroid plexus lining the ventricles and partially from interstitial fluid (ISF) from the brain tissue produced at the BBB. CSF flows through the ventricular system to the subarachnoid space surrounding the brain and spinal cord^5^. Lymphatic vessels have never been identified within the parenchyma of the central nervous system (CNS). Thus, outflow of CSF was historically assumed to directly enter the blood through arachnoid villi that project into the dural venous sinuses^4,6^. However, using a recently developed near-infrared (NIR) imaging method that allowed us to evaluate the dynamics of transport to systemic blood of tracers injected into the lateral ventricle or cisterna magna in mice, we discovered that return of CSF to the systemic blood is predominantly through the lymphatic vasculature in this species. Consistent with several earlier reports^7,8^, CSF tracers exited along perineural routes through foramina of the skull to reach lymphatic vessels outside the cranium^9^.

The outflow of CSF varies between physiological and pathological conditions. As expected from a bulk outflow system, the dynamics of CSF outflow are accelerated under conditions of acute rises in intracranial pressure such as the introduction of large CSF infusion volumes^10–12^. However, it is not yet clear how CSF outflow is altered during pathological conditions, such as the vasogenic edema that occurs in glioblastoma. It is conceivable that similar to the acute conditions, CSF outflow would be accelerated in glioblastoma due to the intracranial hypertension and brain edema. However, edema may alternatively lead to a slower turnover of CSF due to reductions in CSF formation and/or blocked outflow routes. Therefore, it is highly interesting and clinically relevant to study the changes in CSF outflow that occur in glioblastoma. This might potentially lead to novel interventions for brain tumor induced edema, improve the drug delivery in glioblastoma patients and provide insights into glioblastoma antigen drainage.

In this study, we used the GL261 glioma mouse model and employed dynamic NIR imaging and magnetic resonance imaging (MRI) to monitor the CSF distribution and outflow after the infusion of tracers into the cisterna magna. Contrary to our expectations, we found that CSF outflow is significantly reduced in glioma-bearing mice. Dynamic imaging of tracer signals showed that the transport of tracers to the blood was delayed with overall signals at 60 min after tracer infusion significantly lower in glioma-bearing mice. Tracers signals at the perineural outflow routes from the skull were significantly lower in glioma-bearing mice compared to non-tumor bearing mice. By MRI, we found that the tracer in glioma-bearing mice was retained at the cisterna magna injection site compared to non-tumor bearing mice. Surprisingly, in some glioma-bearing mice, we detected high tracer signal in the spinal region. Mice showing high tracer signal in the sacral region of the spine demonstrated lymphatic outflow to iliac lymph nodes and more tracer transport to systemic blood, indicating that CSF may be redirected in glioma-bearing mice to spinal lymphatic outflow pathways after the blockage of cranial CSF outflow routes.

## Results

### Outflow of cerebrospinal fluid is decreased in glioma-bearing mice

**Figure 1:**
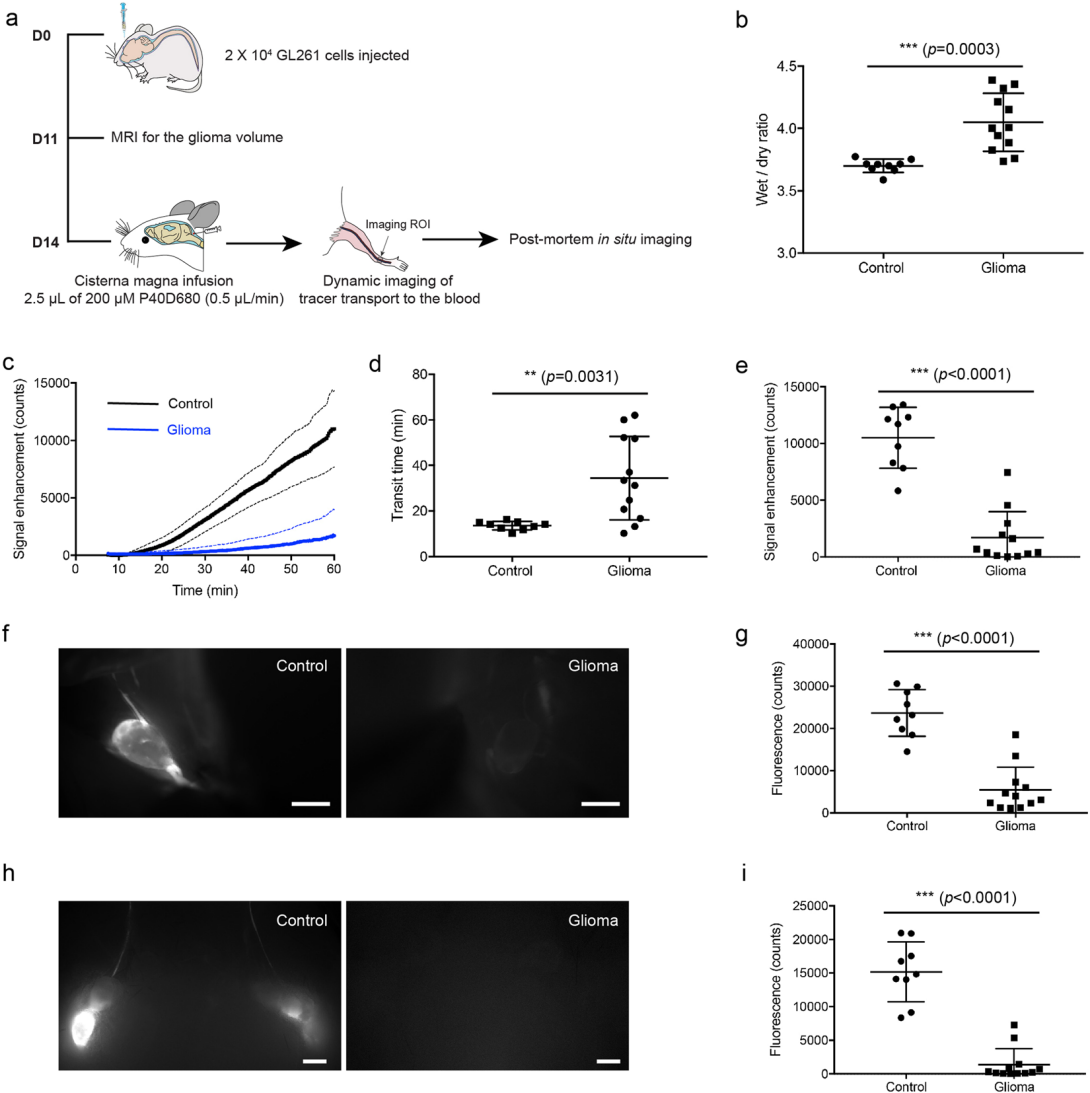
CSF outflow is reduced in glioma-bearing mice. **(a)** Scheme of experiment timeline. **(b)** Edema measure (wet/dry ratio) in controls and glioma mice. **(c)** Saphenous vein signal plot of tracer transport to the systemic blood after cisterna magna infusion of P40D680 (solid line: mean value, dashed line: SD). Control, n = 9; glioma, n = 12. **(d)** Quantification of transit time to the blood after infusion. Data are the mean± SD. **(e)** Tracer signal in the blood 60 min after tracer infusion. Data are the mean± SD. **(f)** Representative images of deep cervical lymph nodes. Scale bar: 1 mm. **(g)** The mean tracer fluorescent signal of deep cervical lymph nodes 60 min after tracer infusion into lateral ventricle. Data are the mean± SD. **(h)** Representative images of mandibular lymph nodes. Scale bar: 1 mm. **(i)** The mean tracer fluorescent signal of mandibular lymph nodes 60 min after tracer infusion into lateral ventricle. Data are the mean± SD.

The first question we addressed was whether there is a change in the CSF outflow to the systemic blood circulation in presence of glioma. For this purpose, we used the GL261 orthotopic xenograft glioma model in C57BL/6 mice (Fig. 1a). An injection of 20,000 GL261 cells in 2 µL was performed into the striatum of the right hemisphere. For the control group, 2 µL PBS were injected at the same location. In glioma-bearing mice, contrast-enhanced MRI was performed 11 days post-inoculation and demonstrated increased vascular leakage in the region of the brain affected by glioma compared to the unaffected hemisphere (Supplementary Fig. 1). We used this property to estimate the glioma volume. Mice with a volume ≤20 mm^3^ were excluded from all further studies. We also confirmed brain edema^13,14^ as indicated by the wet-to-dry weight ratio of the whole brain 14 days after GL261 cell injection. As expected, the degree of brain edema was significantly higher in glioma-bearing brains (4.05 ± 0.23 glioma-bearing mice vs. 3.70 ± 0.054 control, p = 0.0003, unpaired two-tailed t test, Fig. 1b).

At day 14, CSF outflow was measured by monitoring the tracer intensity in the saphenous vein using dynamic NIR imaging up to 60 min after tracer infusion into the cisterna magna^9^. Notably, the transit time to the blood was significantly increased in glioma-bearing mice (34.46 ± 18.28 min glioma-bearing mice vs. 13.64 ± 1.89 min control, p = 0.0031, unpaired two-tailed t test, Fig. 1c,d). The signal enhancement in the blood at 60 min after tracer infusion was 6-fold lower in glioma-bearing mice (1,706 ± 2,284 glioma-bearing mice vs. 10,501 ± 2,687 control, p < 0.0001, unpaired two-tailed t test, Fig. 1e). Next, we measured the tracer signal intensity in CSF-draining lymph nodes. The deep cervical and mandibular (or superficial cervical) lymph node groups, both of which we have previously identified to drain CSF from the cranial cavity^9^, showed significantly lower tracer intensity (deep cervical lymph nodes, 5,444 ± 5,395 glioma-bearing mice vs. 23,662 ± 5,518 control, p < 0.0001, unpaired two-tailed t test, Fig. 1f,g; mandibular lymph nodes, 1,379 ± 2,379 glioma-bearing mice vs. 15,177 ± 4,466 control, p < 0.0001, unpaired two-tailed t test, Fig. 1h,i). These findings indicate that lymphatic outflow of CSF is reduced in the presence of glioma.

### Perineural outflow pathways are not active in glioma-bearing mice

**Figure 2:**
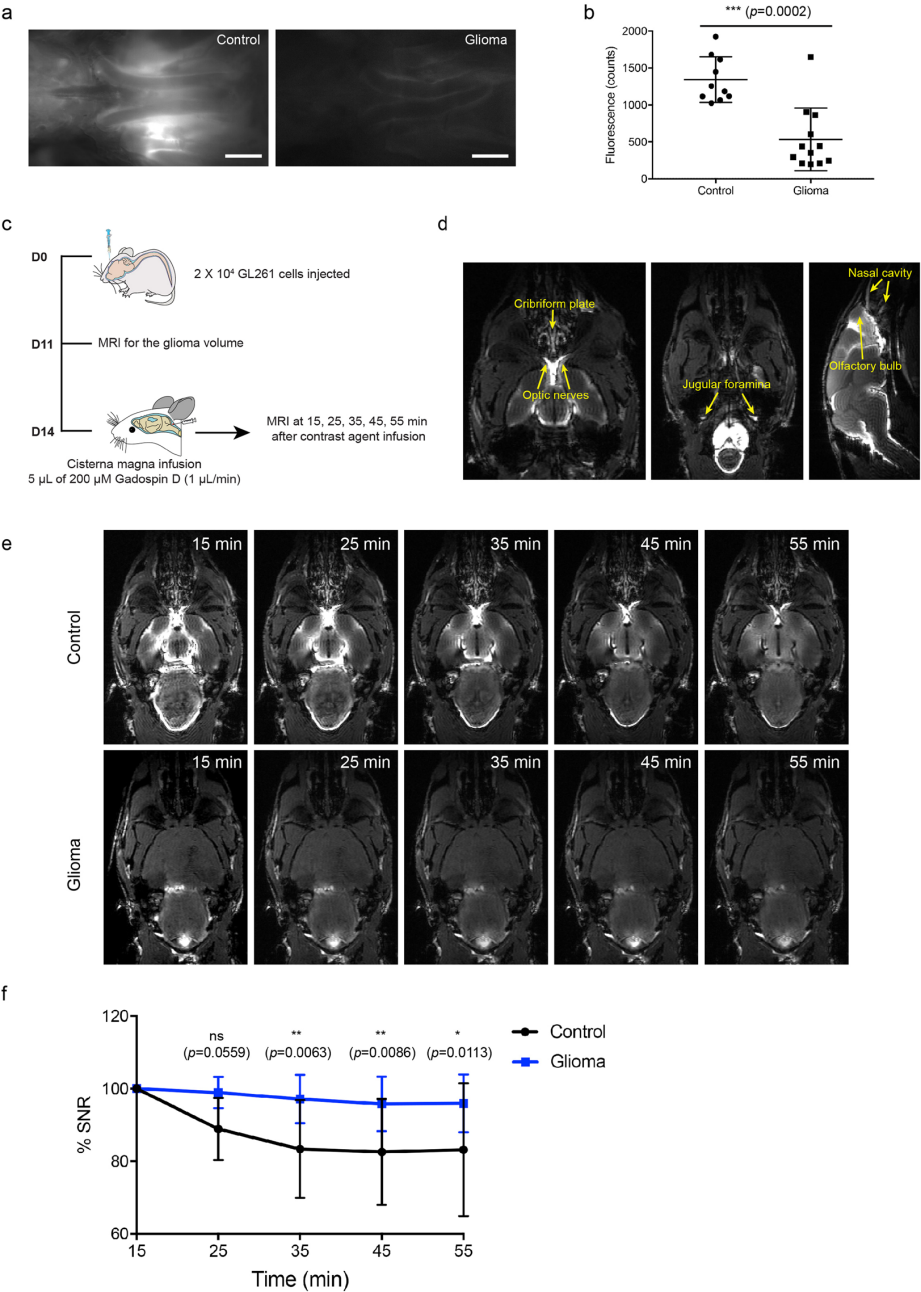
The perineural outflow pathway is not active in glioma-bearing mice. (**a**) Representative images of P40D680 tracer at the optic nerve region in normal and glioma-bearing mice. **(b)** Quantification of tracer signal on the optic nerves. Data are the mean±SD. **(c)** Scheme of experimental timeline. **(d)** Representative MRI images showing the optic nerves, jugular foramina, olfactory bulb and nasal mucosa at 15 min after Gadospin D contrast agent infusion in control mice. **(e)** Representative MRI images of the optic nerves at 15 min, 25 min, 35 min, 45 mins and 55 min after contrast agent infusion. Scale bar: 5 mm. **(f)** Plots of percent change in signal-to-noise ratio (SNR) on optic nerves in control and glioma-bearing mice. The analyzed ROI is shown in Fig. S2. n = 5 in each group. Two-way ANOVA with Sidak’s multiple comparisons test were performed.

As shown in our previous study^9^, macromolecular tracers infused into the CSF in mice are cleared from the subarachnoid space through perineural routes surrounding the exiting cranial nerves of the skull, including olfactory nerves crossing the cribriform plate and optic nerves passing into the orbit. The tracers are then drained by extracranial lymphatic vessels. To test whether these perineural outflow pathways are active in glioma-bearing mice, we measured the tracer intensity in the area over the optic nerves (Fig. 2a) by post-mortem *in situ* imaging at 60 min after tracer infusion. The tracer fluorescence intensity at the optic nerves was about 2.5 times lower in glioma-bearing mice (534.8 ± 426.2 glioma-bearing mice vs. 1,344 ± 307.6 control, p < 0.0001, unpaired two-tailed t test, Fig. 2b). The tracer fluorescence intensity in the lymphatic vessels draining the eye region (the afferent lymphatic vessels of the mandibular lymph nodes, Fig. 1h) was either very low or undetectable. These results indicated that the perineural outflow pathways may be impaired.

To monitor the presence of CSF tracer along the cranial nerves non-invasively over time, we infused Gadospin D, a macromolecular contrast agent, into the cisterna magna 14 days after glioma inoculation and acquired time-of-flight MRI images in horizontal (cranial to dorsal) and sagittal (dorsal to ventral) direction at 15 min, 25 min, 35 min, 45 min and 55 min after infusion (Fig. 2c). Using this method, perineural sites of CSF outflow including optic nerves (CN II), the cribriform plate (CN I) and the jugular foramina (CN IX, CN X, CN XI) were clearly visible in control mice (Fig. 2d). We focused on the optic nerves (region of interest (ROI) shown in Supplementary Fig. 2a) and measured the signal intensity at different time points after infusion (Fig. 2e). This showed that the Gadospin D signal on the optic nerves exhibited a significant decrease over time in control mice, while the signals remained constant in glioma mice (Fig. 2f). Of note, the percent change of the signal to noise ratio (SNR) only gives information of relative intensity changes and does not represent the overall reduced signal on optic nerves that remained much lower even 60 min after cisterna magna infusion in glioma mice as shown by fluorescent imaging (Fig. 2b). Together, these results indicate that the contrast agent that reaches the perineural spaces of the optic nerves after infusion into the cisterna magna is hindered from clearing from this space, further confirming that the normal perineural pathways are not active in glioma-bearing mice.

### CSF clearance from the cisterna magna injection site is reduced in glioma-bearing mice

**Figure 3:**
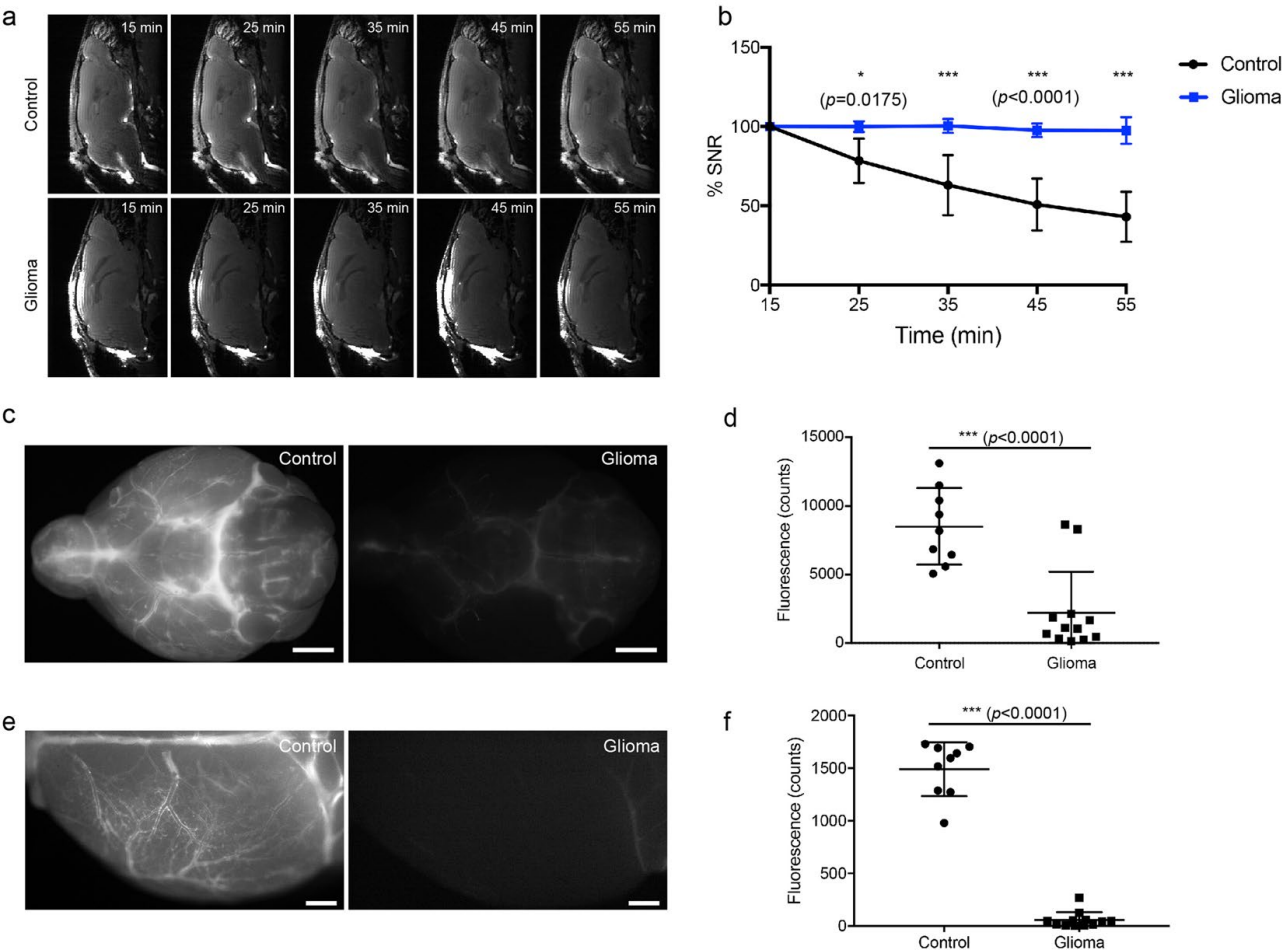
CSF circulation was blocked at the site of the basal cistern. **(a)** Representative MRI images of the cisterna magna at 15 min, 25 min, 35 min, 45 mins and 55 min after contrast agent infusion. **(b)** Plots of percent change in signal-to-noise ratio (SNR) at the cisterna magna. The analyzed ROI is shown in Fig. S2. n = 5 in each group. Two-way ANOVA with Sidak’s multiple comparisons test was performed. **(c)** Representative images of P40D680 tracer at the circle of Willis (basal cistern region) in normal and glioma-bearing mice. Scale bar: 2 mm. **(d)** Quantification of tracer signal at the basal cistern region. Data are the mean± SD. **(e)** Representative images of P40D680 tracer at the brain surface of the left dorsal hemisphere in normal and glioma-bearing mice (contralateral to the glioma site). Scale bar: 1 mm. **(f)** Quantification of tracer signal on the dorsal brain surface. Data are the mean± SD.

To further study the concept of a reduced CSF circulation within the cranial subarachnoid space, we next measured the contrast agent intensity at the cisterna magna over time using MRI (Fig. 3a). Here, we found that the contrast agent intensity at the cisterna magna continuously decreased in the control group while it remained relatively constant in the glioma group (Fig. 3b). This indicates that under normal conditions, contrast agent infused into the cisterna magna is constantly distributed to the subarachnoid space via CSF circulation, whereas in glioma-bearing mice the spread of contrast agent was inhibited. From the cisterna magna, a bulk flow of CSF reaches the cisternal spaces on the basal aspect of the brain along the circle of Willis. Depending on the physiological conditions, from this location CSF either exits the skull along the perineural spaces or distributes in a paravascular fashion over the dorsal aspect of the brain along the superficial arteries of the brain^15^. To map out the tracer distribution within CSF, we perfused P40D680 NIR tracer into the cisterna magna and imaged *ex vivo* the basal aspect of the brain and the dorsal surface of the hemisphere contralateral to the glioma/PBS inoculation side of the brain. The tracer intensity in the basal aspect of the brain was 3 times weaker in the glioma mice compared to controls (2,209 ± 2,998 glioma-bearing mice vs. 8,496 ± 2,784 control, p < 0.0001, unpaired two-tailed t test, Fig. 3c,d), and the difference on the dorsal brain surface was even greater, almost 7 times weaker in the glioma group compared to controls (56.79 ± 74.98 glioma-bearing mice vs. 1,491 ± 256.8 control, p < 0.0001, unpaired two-tailed t test, Fig. 3e,f). Together, these data show that in glioma-bearing mice, the normal pathways of CSF circulation were inhibited, with reduced tracer flow from the cisterna magna to the basal aspect of the brain and to the paravascular spaces of the dorsal brain surface.

### CSF is redirected to the spinal cord in glioma-bearing mice

**Figure 4:**
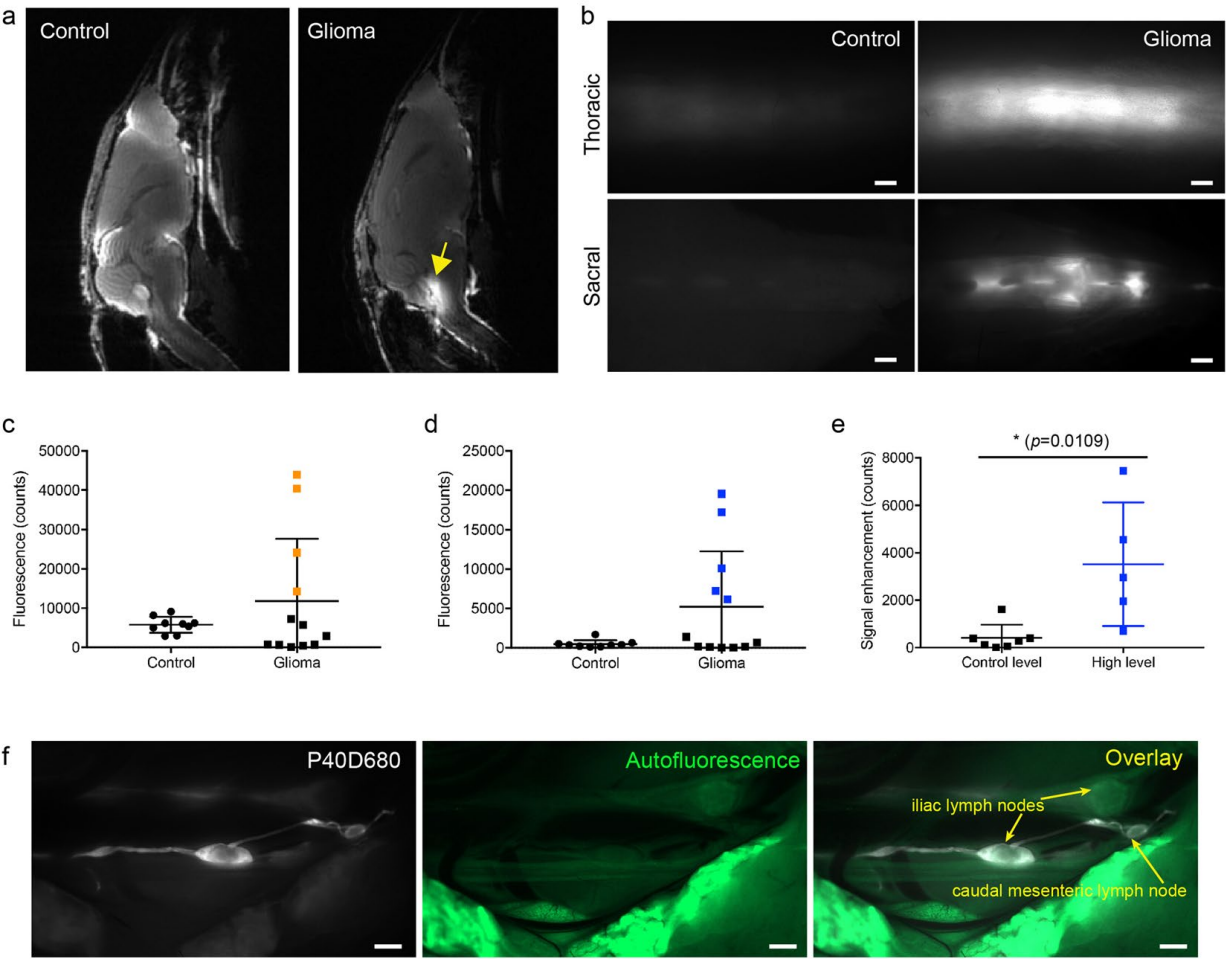
CSF is redirected to the spine and spinal lymphatic outflow pathways in glioma-bearing mice. (**a**) Representative MRI images showing the distribution of contrast agent distribution at 55 min after cisterna magna infusion. The fourth ventricle is indicated by a yellow arrow. **(b)** Representative images of the thoracic and sacral region of the spine in control mice and glioma-bearing mice which showed increased tracer signal. Scale bar: 1 mm. Quantification of tracer signal in the thoracic **(c)** and sacral region **(d)** of the spine. Data are the mean± SD. **(e)** Comparison of tracer levels in the systemic blood at 60 min in the glioma group between mice with high sacral signals (blue squares in d) and control-level sacral signals (black squares in d). Data are the mean± SD. **(f)** Representative image of the iliac and caudal mesenteric lymph nodes in glioma-bearing mice with sacral lymphatic outflow. Scale bar: 1 mm.

CSF circulates within the subarachnoid space around both the brain and the spinal cord^16^. Previous studies have shown that when the circulation of CSF within the cranium is blocked, such as in the kaolin-induced hydrocephalus model, more CSF flow may be redirected towards the spinal cord^17^. Bearing this in mind, we studied MRI scans at 55 min after tracer infusion (Fig. 4a). In the control group, the contrast agent was distributed throughout the cranial cavity, with limited spread towards the spinal cord. In glioma-bearing mice, the signal of the contrast agent was much less apparent in the cranial space and instead was clearly visible in the subarachnoid space of the upper spinal column. In glioma-bearing mice, there was often contrast agent signal in the fourth ventricle (Fig. 4a, indicated by yellow arrow), indicating that CSF blocked at the cisterna magna might be redirected towards the ventricles.

We have recently shown that tracer distribution within the SAS of the spine at 60 min after cisterna magna infusion is limited to the thoracic region with typically no evidence of tracers at the sacral spine^16^. Thus, using NIR imaging in glioma-bearing and control mice, we quantified the tracer distribution in the thoracic and sacral regions of the spinal cord at 60 min after infusion (Fig. 4b). We found that in 4 out of 12 glioma-bearing mice, the signal intensity in the thoracic region was increased above the levels of the control mice (indicated in orange, Fig. 4c), and 5 out of 12 mice exceeded the signal intensity range of control mice in the sacral region (indicated in blue, Fig. 4d). Only one mouse showed increased signal in both the thoracic and sacral spinal regions. Thus, overall 8 out of 12 glioma-bearing mice demonstrated abnormally high tracer signals in the spine. Interestingly, lymphatic outflow of the tracers was only observed in the glioma-bearing mice with increased signals in the sacral region, consistent with our concept that this region is the major outflow site to lymphatics from the spine^16^. Tracers were found within lymphatic vessels leading to the iliac or/and caudal mesenteric lymph nodes^18^ (Fig. 4f). Furthermore, in mice with increased sacral spinal signals, the tracer levels in the systemic blood detected by dynamic NIR imaging were also significantly higher, indicating lymphatic transport from the spinal outflow site to the blood (3,523 ± 2610 mice with high sacral signals (blue squares in Fig. 4d) vs. 408.9 ± 522.4 control-level sacral signals (black squares in 4d), p = 0.0109, unpaired two-tailed t test, Fig. 4e). This compensatory CSF transport through the spine to blood in mice bearing glioma reached up to 70% of normal CSF outflow levels. Unexpectedly, the two mice with the highest spinal outflow compensation presented with scoliosis with a curved spine (Supplementary Fig. 3), indicating that such rerouting might exert adverse effects. In summary, these results indicate that CSF flow was redirected to the spinal cord when the spread towards cranial outflow routes was blocked. Thus, lymphatic outflow from the spinal cord might serve as a compensatory route for CSF outflow in glioma conditions.

## Discussion

One might expect that CSF outflow would increase during glioblastoma development, as high intracranial pressure might appear to be a powerful drive for fluid to exit the skull. However, this concept is not in line with several clinical observations, including the prevalence of persistent brain edema, the rare occurrence of extracranial metastasis in glioblastoma patients^19^ and the limited anti-tumor immune response in glioblastoma^20^. In this study, by dynamic NIR imaging and MRI of tracer distribution after cisterna magna infusion, we have elucidated that the outflow of CSF is decreased in glioma-bearing mice.

We previously demonstrated that outflow of CSF occurs predominantly through lymphatic vessels, and by dynamic imaging of the posterior facial vein that collects blood from the transverse sinus in mice, we excluded a direct route from CSF to blood^9^. In the current study, we provide more support for this concept in pathological conditions. We found - using NIR dynamic imaging in glioma-bearing mice - that after cisterna magna infusion, the transport of tracer to the blood showed a delay before signal enhancement, which is the typical pattern for lymphatic outflow^21^. Glioblastoma is highly vascularized, the new blood vessels formed during tumor-associated angiogenesis are usually leaky, and the BBB is often disrupted with tumor progression^22^. Our findings demonstrate that despite an extended tumor-associated blood vessel network with leakiness and edema formation, CSF fluid outflow in glioma-bearing mice still proceeds through lymphatic vessels. This emphasizes the importance of lymphatic outflow in both physiological and pathological conditions of the central nervous system. Cervical lymph nodes are one of the most commonly reported locations of extracranial glioblastoma metastases^23–25^, which could also serve as anecdotal clinical evidence that CSF outflow is through lymphatic vessels under these conditions.

In most cancer types, tumor expansion occurs within tissues with high compliance. Blood vessel angiogenesis and increased vascular leakage lead to an increased lymphatic flow from the tumor site^26,27^. Lymphangiogenesis is then induced to cope with the increased fluid load^28^. However, the situation in glioblastoma is unique since the tumors develop within the cranial cavity with a defined volume and within a tissue in which lymphatic vessels are not present. As the tumors expand, the glioblastoma and its associated edema cause the brain tissue to occupy more intracranial space, including the subarachnoid space, which might obstruct the CSF circulation. Our imaging data showed that in glioma-bearing mice, the tracer signal at the site of injection remained almost unchanged, and the tracer signal intensity at the basal aspect of the brain and along the cranial nerves was very low, indicating that CSF circulation from the site of the cisterna magna was blocked. The expression of the lymphangiogenic factor VEGF-C has been reported to be very low in glioblastoma^29^, which may also hinder the expansion of lymphatic vessels that are draining the CNS. Thus, unlike peripheral tumors, fluid outflow in glioblastoma appears to be reduced. This finding likely has clinical relevance as it has been found that a certain percentage of glioblastoma patients develop hydrocephalus, and shunting of CSF from the ventricles to the peritoneal cavity has recently been employed to alleviate the symptoms in these patients^30,31^.

A reduced fluid turnover in glioblastoma may have important implications for disease progression. Although not directly assessed in this study, CSF production has also been reported to be reduced with brain edema and high intracranial pressure^32–34^. Decreased production together with impaired outflow largely reduce the turnover rate of brain fluid, which may enable the accumulation of toxic proteins, solutes, pro-inflammatory cytokines and chemokines. This chronic inflammatory status might accelerate glioblastoma progression. With reduced CSF outflow, transport of glioblastoma-derived antigen to draining lymph nodes is also likely be minimal, compromising the activation and proliferation of anti-tumor T cells, which offers glioblastoma a privileged environment to progress. The disrupted CSF circulation would also attenuate the efficacy of intrathecal drug delivery to the cranial cavity.

We have previously found, using a primary cutaneous melanoma model, that upon blockage of the normal lymphatic drainage route due to growth of metastases within the downstream lymph nodes, the lymphatic network could reroute to bridge the blockage and restore the drainage^35^. Similarly, in lymphedema models, collateral flow routes are also utilized to maintain lymphatic drainage after the resection of draining lymph nodes^36^. Interestingly, we also observed a “rerouting” of CSF outflow to the spinal column in the glioma model. We have recently identified lymphatic outflow pathways from the sacral region of the spine of mice that are most apparent after infusion of tracers into the lateral ventricle, indicating a caudal flow of CSF in the central canal^16^. However, outflow from the spine appears to be minor in comparison to the cranial outflow routes under normal conditions. In glioma-bearing mice, there was an increased distribution of tracer in the spine after cisterna magna infusion with significant lymphatic outflow from the sacral region in some mice. Compensation of outflow of CSF to the spinal space was described as early as 1901 by Theodor Kocher as a response to an intracranial expanding lesion^37^. A rerouting of flow into collateral pathways down the spine, such as the central canal, has been reported in many hydrocephalus models^38–40^. Similar to our results, in a kaolin-induced model of hydrocephalus in rats, this collateral flow could be followed to the spine-draining nerve roots and extradural lymphatics, as evidenced by ferritin tracing^17^. However, extensive rerouting to the spine may not be desirable as we observed that the two mice which exhibited the highest lymphatic compensation from the spine also had developed scoliosis. It has been previously reported in kaolin-induced hydrocephalus models that obstructed CSF outflow from the skull can lead to abnormalities in the spine including syringomyelia and scoliosis^41,42^, which is in agreement with our observation. Although it was not observed in our acute mouse model, a rerouting of CSF flow down the spine may also contribute to spinal metastases^43–45^. Initially thought to be a rare occurrence, the prevalence of spinal metastases in the clinic has become increasingly apparent with improved MR imaging protocols and more awareness of this phenomenon^43^.

In conclusion, our study reveals that outflow of CSF is reduced in glioma due to a blockage of CSF circulation in the cranial cavity. CSF is redirected into the spinal space and the lymphatic outflow from the spinal space can partially compensate for the impaired CSF outflow. The reduced CSF outflow might intensify the brain edema, aggravate the brain tumor microenvironment and attenuate intracranial drug delivery efficacy. Thus, treatments and procedures that can potentially compensate CSF outflow and restore normal CSF circulation are worthy of clinical attention.

## Materials and Methods

### Mice

Female wild-type mice (Janvier Labs) and Prox1-GFP reporter mice^46^ on the C57BL/6 J background were kept under specific pathogen-free conditions and used for experimental studies at the age of 2 to 3 months.

### Cell culture

GL261 cells were maintained in DMEM supplemented with l-glutamine, 10% FBS, and penicillin/streptomycin under standard culture conditions (37 °C, 5% CO_2_)^47^.

### Intracranial implantation of GL261 cells

C57BL/6J mice were subcutaneously injected with 0.1 mg/kg buprenorphine (Temgesic, Reckitt Benckiser AG, Switzerland) 30 mins before the procedure. The mice were anesthetized by inhalation of 2.5% isoflurane and fixed in a stereotaxic frame (RWD, San Diego, CA) on a heating pad (37 °C). The skull was thinned with a dental drill (RWD) at a location 2 mm lateral and 2.5 mm caudal to the bregma. A single cell suspension of 20,000 GL261 cells was prepared in 2 µl of PBS and injected at a speed of 1 μL/min with a 26 G needle into the right striatum at a depth of 3 mm from the skull surface. The needle was left in place for 2 minutes and then slowly removed while observing if any significant backflow occurred. The injection hole was closed with bone wax (ETHICON) and the scalp was sutured and glued with tissue glue (Histoacryl). The mice were observed in a cage with a heating pad at 37 °C until fully recovered from anesthesia.

### Infusion of CSF bulk flow tracers into the cisterna magna

Mice were anesthetized by intraperitoneal injection of 80 mg/kg ketamine and 0.2 mg/kg medetomidine, fixed in a stereotaxic frame (RWD) and maintained at 37 °C body temperature using a heating pad (Stoelting). The injection was performed as described in a previous study^15^. A skin incision was made over the occipital bone and the covering muscle layers were carefully dissected to expose the atlanto-occipital membrane covering the cisterna magna. A glass capillary with a diameter of 30 ~ 50 µm was used to penetrate the membrane perpendicular to the dura. For the MRI experiments, 5 µL Gadospin D solution (25 mM Gadolinium, Viscover) was infused at the speed of 1 µL/min. For the NIR imaging experiments, 2.5 µL of 200 µM P40D680 was infused at the speed of 0.5 µL/min. After the infusion, the capillary was left in place for 5 min and tissue glue (histoacryl) was applied at the injection site. The tip of the glass capillary was then cut off by spring scissor and the wound was closed by tissue glue.

### Dynamic NIR imaging of CSF outflow

For noninvasive imaging of tracer signals in the blood^21^, fur above the saphenous vein region was removed with a razor and depilation cream before the tracer infusion. After closure of the wound after tracer infusion, the mice were immediately positioned under a Zeiss StereoLumar.V12 stereomicroscope with AxioVision software (Carl Zeiss, Feldbach, Switzerland) and a Photometrics Evolve 512 camera (Photometrics, Tuscon, AZ) in a supine position on a heating pad to retain body temperature. The autofluorescence signal on the GFP channel was used to position the saphenous blood vessels at 25× zoom. Dynamic imaging was initiated 10 minutes after completion of the cisterna magna infusion by acquisition of a sequence of images (1 image every 15 s for 50 min) with a Cy5 filter set to monitor the NIR signal of the saphenous vein. Exposure time and camera gain settings were 200 ms and 200, respectively.

To assess the tracer transport to the systemic blood, a circular region of interest with a radius of 100 μm was placed over the saphenous vein on the acquired videos. Using the Measure Profile function, a table of fluorescence intensity in counts versus time was exported into Microsoft Excel. Since there was a loss of signal at the beginning of the scans due to photobleaching of tissue autofluorescence, baseline intensity in counts was calculated as the average signal of the lowest ten consecutive imaging frames. This baseline intensity was then subtracted from the fluorescence intensity values in order to plot fluorescent signal enhancement versus time in minutes. The NIR fluorescent signal enhancement value in counts at t = 60 min and the transit time in minutes of the arrival of tracer to the blood circulation (set at a threshold of 100 counts of signal enhancement) were assessed.

### Analysis of post-mortem NIR tracer distribution

Images of P40D680 tracer spread on the basal and dorsal aspects of the brain, within the CNS-draining lymph nodes and within the spinal column were acquired with a Zeiss AxioZoom V16 microscope and a QImaging OptiMOS sCMOS camera (QImaging, Surrey, Canada) combined with a light-emitting diode illumination system pE-4000 (CoolLED Ltd, Andover, UK) and ZEN 2 software (Carl Zeiss, Feldbach, Switzerland). In excised brains, images were acquired over the contralateral dorsal hemisphere (20×, 20 ms exposure) and on the entire ventral side of the brain (11.2×, 200 ms exposure). Images of lymph nodes were acquired *in situ* at 25× and 200 ms exposure time. Since there were no apparent differences in signal in the lymph nodes on the injected and contralateral sides, the average value of the nodes on each side was used for statistical analysis. The thoracic and sacral regions of the spine were imaged after removal of the skin at 15× and 200 ms exposure time. The region of the thoracic image starts from the junction point of the muscle spinalis et semispinalis thoracis and muscle longissimus thoracis and extends in a caudal direction with the spine in the middle of the image. The region of the sacral region image starts from the base of the tail and extends in a rostral direction with the spine in the middle. For analysis of signal enhancement in the analyzed tissues, background signals were subtracted based on the average signal of 3 uninjected mice.

### Magnetic resonance imaging of CSF dynamics

To measure the contrast agent distribution after cisterna magna infusion, mice were anesthetized and 5 µL Gadospin D (25 mM gadolinium, Viscover) infused at a speed of 1 µL/min. Immediately after injection, the head of the mouse was fixed in the stereotactic frame attached to a 9.4 T animal scanner (Bruker Biospin GmbH) with a BGA12S gradient system with ParaVision 6.0.1 (Bruker Biospin GmbH). A four–element receive–only cryogenic phased array coil (Bruker BioSpin GmbH) was used in combination with a linearly polarized room temperature volume resonator for transmission. Imaging was performed with a three-dimensional time of flight gradient recalled echo sequence (3D-TOF-GRE)^48^ with an echo time/repetition time = 2.5/12.0 ms, flip angle 25°, matrix 480 × 360 × 180, field of view 36 mm × 27 mm × 18 mm, zero fill 2, 1 average and a scan time of 3 min 46 s. Both horizontal (dorsal to ventral) and sagittal (rostral to caudal) sections were scanned at t = 15 min, 25 min, 35 min, 45 min, 55 min after Gadospin D infusion. Signal intensity (SI) reduction in blood vessels was achieved by placement of a saturation slice over the mouse heart. SI and noise were measured with ImageJ (Version 2.0.0). The ROI placements and the signal over noise ratio calculation were shown in Fig. S2.

### Magnetic resonance imaging of glioma volume

To determine the glioma volume, glioma-bearing mice were anesthetized by inhalation of 2.5% isoflurane and injected with the contrast agent Dotarem (1.5 µL/g, Guerbet, 0.5 mmol acidum gadotericum) subcutaneously 5 mins before imaging. The measurement was performed in a 7 T animal magnetic resonance imaging (MRI) system (Bruker BioSpin GmbH). A four–element receive–only cryogenic phased array coil (Bruker BioSpin GmbH) was used in combination with a linearly polarized room temperature volume resonator for transmission. The mice were stereotactically fixed and the body temperature maintained at 37 °C. A rapid acquisition with relaxation enhancement (2D TurboRARE) sequence was used with the following parameters: echo time/ repetition time = 36/2200 ms, rare factor = 8, BW = 50 kHz, FOV 15 mm × 15 mm, matrix 256 × 256, slice thickness = 0.5 mm, 13 slices, 3 averages, scan time = 2 min 38 s. The scans were analyzed with ImageJ (Version 2.0.0). The volume of the glioma was calculated as the sum of glioma area in each slide multiplied by the slide thickness.

### Statistical analyses

Statistical analyses were performed with GraphPad Prism5 (GraphPad Software Inc.). Graphs represent mean ± SD. Student’s t-test was used to compare two groups. Other types of tests are indicated in the figure legends. A p-value < 0.05 was considered statistically significant (indicated by asterisks).

### Ethical approval

All mouse experiments were approved by the Kantonales Veterinaramt Zurich (license numbers 185/13 and 161/16) and performed following the regulations of the Swiss Federal Welfare Act (TSchG).

## Supplementary information

Supplementary Information

## Data Availability

All data needed to evaluate the conclusions in the paper are present in the paper and/or the Supplementary Materials. Additional data related to this paper may be requested from the corresponding authors on reasonable request.
