# Supplementary material for: Lymphatic outflow of cerebrospinal fluid is reduced in glioma
Source: Sci Rep. 2019 Oct 15;9:14815. doi: 10.1038/s41598-019-51373-9 (PMC6794292; doi:10.1038/s41598-019-51373-9)
Supplement: Supplementary file 1 — Supplementary Information [file 41598_2019_51373_MOESM1_ESM.pdf]

Supplementary Information

Lymphatic outflow of cerebrospinal fluid is reduced in glioma

Qiaoli Ma[1], Felix Schlegel[2,3], Samia B. Bachmann[1], Hannah Schneider[4], Yann Decker[5],

Markus Rudin[2,3,6], Michael Weller[4], Steven T. Proulx[1,7*], Michael Detmar[1*]

[1]Institute of Pharmaceutical Sciences, Swiss Federal Institute of Technology, ETH Zurich, Zurich, Switzerland

[2]Institute of Biomedical Engineering, University of Zurich and ETH Zurich, Zurich, Switzerland

[3]Neuroscience Center Zurich, University of Zurich and ETH Zurich, Zurich, Switzerland

[4]Laboratory of Molecular Neuro-Oncology, Department of Neurology, University Hospital and University of Zurich, Zurich, Switzerland

[5]Department of Neurology, University of the Saarland, Homburg, Germany

[6]Institute of Pharmacology and Toxicology, University of Zurich, Zurich, Switzerland

[7]Theodor Kocher Institute, University of Bern, Bern, Switzerland

[*]Corresponding authors

Email: michael.detmar@pharma.ethz.ch, steven.proulx@tki.unibe.ch

Supplementary figures

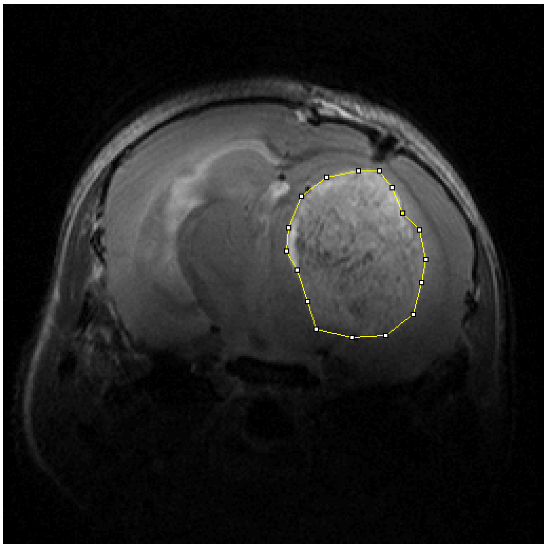

Fig. S1. Representative image of contrast-enhanced MRI for glioma volume measurement on day 11 after GL261 cell injection. Yellow line indicates the margin of the glioma indicated by the contrast agent.

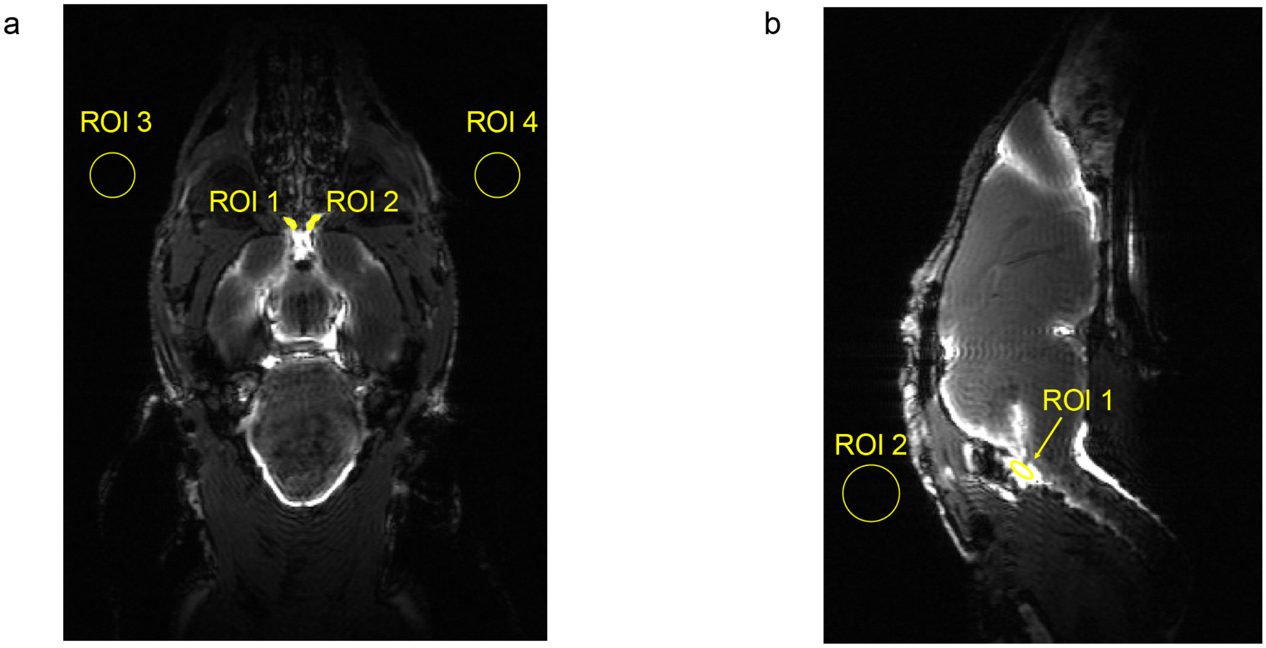

Fig. S2. ROIs for SNR calculation. (**a**) Optic nerve. SNR= (Mean Signal Intensity$_{ROI1}$/SD$_{ROI3}$ +Mean Signal Intensity$_{ROI2}$/SD$_{ROI4}$)/2. (**b**) Cisterna magna. SNR=Mean Signal Intensity$_{ROI1}$/SD$_{ROI2}$.

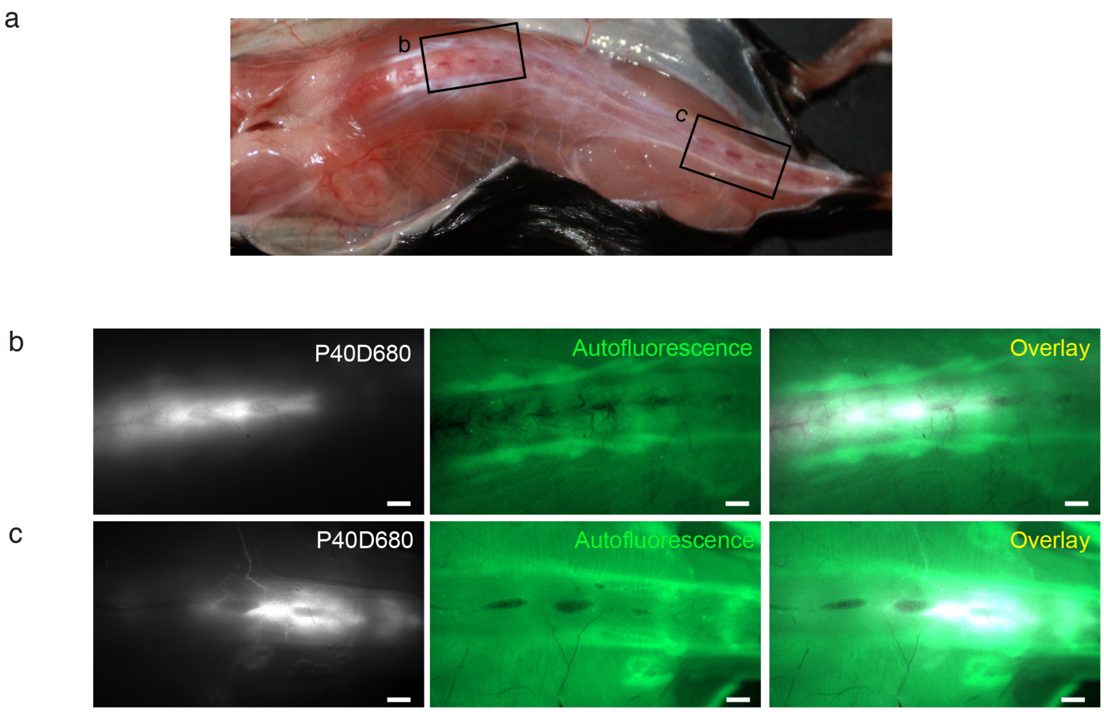

Fig. S3. Increased tracer signal in spine and scoliosis. (**a**) Bright field image of spine of a mouse with scoliosis symptom. Fluorescent images of thoracic (**b**) and sacral region (**c**) of the spine. Scale bar: 1000 μm.
